# Glycodeoxycholic acid synergizes with L-malic acid to upregulate the malolactic enzyme pathway to alleviate self-toxicity in *Lacticaseibacillus paracasei* L9

**DOI:** 10.1128/aem.00639-26

**Published:** 2026-06-18

**Authors:** Ran Huan, Xin Feng, Yaxin Gu, Junzhu Li, Jianping Cai, Zhengyuan Zhai, Yanling Hao

**Affiliations:** 1 Department of Basic Innovation Research, The Key Laboratory of Geriatrics of NHC, Beijing Key Laboratory of Aging Mechanism and Intervention Research on Aging-Related Diseases, Beijing Hospital, National Center for Gerontology, National Clinical Research Center for Gerontology, Institute of Geriatric Medicine, Chinese Academy of Medical Sciences 665500, Beijing, China; 2 College of Food Science and Nutritional Engineering, China Agricultural University 34752, Beijing, China; 3 Key Laboratory of Precision Nutrition and Food Quality, Department of Nutrition and Health, China Agricultural University 34752, Beijing, China; Universita degli Studi di Napoli Federico II, Portici, Italy

**Keywords:** GDCA stress, malolactic enzyme pathway, *Lacticaseibacillus paracasei* L9, bile salt tolerance

## Abstract

The malolactic enzyme (MLE) pathway induced by L-malic acid plays an important role for *Lacticaseibacillus paracasei* L9 to enhance the glycodeoxycholic acid (GDCA) tolerance. However, whether there exists a synergistic effect between L-malic acid and GDCA to cope with bile salt stress remains unclear. In this study, when *Lc. paracasei* L9 was grown in chemically defined medium (CDM) with 0.08% GDCA and 5 g/L L-malic acid, real-time quantitative PCR results demonstrated that the expression of *mleS* (the malolactic enzyme) and the *mleT* (L-malic acid transporter) genes was upregulated by 2.53-fold and 3.63-fold higher than that of the bacteria only cultured in 5 g/L L-malic acid. Furthermore, transcriptional fusion experiments confirmed that GDCA could synergistically enhance the expression of the MLE pathway in the presence of L-malic acid by comparing the intensity of far-red fluorescent protein. Moreover, in the presence of GDCA, the fluorescence intensity increased gradually with increasing L-malic acid concentration, reaching the highest level at 2.0 g/L of L-malic acid, suggesting that GDCA synergistically enhanced the MLE pathway expression in a dose-dependent manner with L-malic acid. The high-performance liquid chromatography (HPLC) quantitative analysis demonstrated that GDCA effectively enhanced the MLE pathway by increasing the intracellular transportation of L-malic acid. Finally, the tolerance ability of the *mleR* (MLE regulator)-deleted strain to bile salt was significantly decreased, which further revealed that GDCA synergized with the MLE pathway to relieve self-toxicity for *Lc. paracasei* L9. These results will lay the foundation for a systematic understanding of the bile salt tolerance mechanism of lactic acid bacteria.

**IMPORTANCE:**

Tolerance to bile stress is critical for probiotics to survive in the gastrointestinal tract. However, the synergistic regulation of stress response pathways by bile salts and metabolic inducers remains unclear. This study elucidates a novel mechanism by which glycodeoxycholic acid (GDCA) cooperates with L-malic acid to upregulate the malolactic enzyme (MLE) pathway in *Lacticaseibacillus paracasei* L9. Specifically, GDCA significantly enhances *mleS* and *mleT* gene expression in the presence of L-malic acid, and this synergistic effect exhibits a dose-dependent relationship with the L-malic acid. Mechanistically, GDCA promotes intracellular L-malic acid transport, thereby increasing the availability of the MleR effector to activate pathway transcription. Consequently, deletion of *mleR* significantly reduces the bile salt tolerance of L9, confirming the essential role of this regulatory circuit. These findings reveal how lactic acid bacteria transform membrane damage into a metabolic defense advantage, providing new insights into adaptive strategies for multi-stress environments.

## INTRODUCTION

*Lacticaseibacillus paracasei* L9 is an aerotolerant Gram-positive bacterium belonging to the homofermentative lactic acid bacteria, which has been extensively used as a probiotic for human health and in fermented dairy. *Lc. paracasei* L9 has many health-promoting properties, including alleviating allergic responses (1), preventing diarrhea (2), regulating oral and gut microbiota (3, 4), ameliorating obesity (5), and enhancing innate immunity (6). However, during consumption, *Lc. paracasei* L9 will suffer from bile salt stress in the intestine. Bile salt exists as glycine or taurine derivatives in the human intestine, mainly including glycodeoxycholic acid (GDCA), glycocholic acid (GCA), taurodeoxycholic acid (TDCA), and taurocholic acid (TCA), which can disrupt the lipid bilayer structure of cellular membranes, cause DNA damage, intracellular acidification, and result in cell death (7–9). Therefore, tolerance to bile stress is critical for *Lc. paracasei* L9 to survive in the gastrointestinal tract (GIT).

Indigenous lactobacilli in the GIT have employed several types of resistance mechanisms to cope with the toxic effects of bile (8, 10). Bile salt hydrolase can catalyze glycine- or taurine-conjugated bile salt into unconjugated counterparts (11). Bile efflux pump is another prevalent mechanism mediated by multidrug resistance transporters, which can extrude the bile salt from the cell (12). In addition, some lactobacilli strains modulate the fatty acid composition and membrane proteins of the cell envelope to decrease membrane permeability in response to bile salt (13–15). Our previous research found that the malolactic enzyme (MLE) pathway could enhance GDCA tolerance in the GIT of *Lc. paracasei* L9 (16). The MLE pathway comprises a malolactic enzyme MleS and an L-malic acid transporter MleT (17). The MleT transports extracellular L-malic acid into bacterial cells in the form of anions (18). L-malic acid is subsequently catalyzed by MleS into L-lactic acid and CO_2_, and an intracellular H^+^ is utilized during the reaction (19). This process prevents the acidification of the growth medium, reduces the toxicity of bile salt to bacterial cells, and maintains cellular morphology, thereby protecting bacterial cells from bile salt stress-induced damage (16, 18, 20). However, it remains unclear whether there is a synergistic relationship to upregulate the MLE pathway between bile salt and inducer L-malic acid.

In this study, gene expression analysis and transcriptional fusion experiments reveal that GDCA and L-malic acid synergistically enhance the expression of the MLE pathway. Furthermore, GDCA can enhance the expression of the MLE pathway by increasing the cytosolic L-malic acid concentration. These results provide insight into the mechanism underlying the MLE pathway in response to bile salt stress.

## MATERIALS AND METHODS

### Bacterial strains, plasmids, and culture conditions

All bacterial strains and plasmids used in this study are listed in Table 1. *Lc. paracasei* L9 cells were incubated statically and anaerobically at 37°C in chemically defined medium (CDM) (21). *Lactococcus lactis* NZ9000 was incubated statically and anaerobically at 30°C in M17 medium containing 0.5% (wt/vol) glucose (GM17). When necessary, the medium was supplemented with 10 µg/mL erythromycin for both *L. lactis* NZ9000 and *Lc. paracasei* L9.

**TABLE 1:** Bacterial strains and plasmids used in this study

Strain or plasmid	Relevant characteristics	Source or reference
Strains		
*Lc. paracasei* L9	Host strain, isolated from the feces of a healthy donor	Laboratory collection
*L. lactis* NZ9000	Plasmid-free derivative of *L. lactis* MG1363 *pepN::nisRK*	Laboratory collection
*ΔmleR*	*mleR* gene deletion mutant of *Lc. paracasei* L9	This work
Plasmids		
pNZ8148EM	pNZ8148 derivative carrying Em^R^ instead of Cm^R^	Laboratory collection
pNZ-Farred	pNZ8148 derivative carrying Em^R^ instead of Cm^R^; P*nisA::Farred* gene in pNZ8148EM, Em^R^	This work
pNZRS	Transcriptional level reporter vector; *P_mleST_*::*far-red* gene in pNZ8148EM, Em^R^	This work

### Quantitative real-time reverse transcription-PCR

Overnight cultures of *Lc. paracasei* L9 wild-type and the *mleR*-deleted strain (*ΔmleR*) were inoculated into CDM, CDM + 0.08% GDCA, CDM + 5 g/L L-malic acid (CDMM), and CDM + 5 g/L L-malic acid + 0.08% GDCA (CDMM + 0.08% GDCA) at 37°C, respectively. When the cell was grown at 37°C for 14 h, 2 mL aliquots were collected from each culture and centrifuged at 8,000 × *g* for 5 min. The total RNA was extracted using an RNAprep Pure Cell/Bacteria Kit (Tiangen Biotech, Beijing, China). Subsequently, the cDNA was synthesized from 500 ng of total RNA using the HiScript III RT SuperMix for qPCR (Vazyme Biotech, Nanjing, China). Quantitative real-time reverse transcription-PCR (RT-qPCR) was performed using the SYBR Green qPCR master mix + genomic DNA (+gDNA) wiper (Vazyme Biotech, Nanjing, China), with an ABI StepOne Plus real-time PCR system (Applied Biosystems Inc.). The primers used for RT-qPCR analysis are listed in Table 2. The expression of the 16S rRNA gene was used as the reference gene. The fold changes in the level of gene expression were calculated using the ΔΔCT method (22).

**TABLE 2:** Primers used in this study

Primer	Sequence (5′−3′)
UP-F	CTCTTCTGCAGGTCGACTCTAGATGGACAAGCTGAATGATGAAACC
UP-R	AGGTAACACTCGCTGTCATGTCAGTCGTG
Down-F	CATGACAGCGAGTGTTACCTCCATGAAACGG
Down-R	AAAACGACGGCCAGTGAATTCGCCGATTCCCAAGATGCC
mleR-UR	CGTGATGGCCGGCCTTAA
mleR-DR	AACCGGGAGAACTTGACT
mleR-UF	GATCACGATTACCCACGATC
mleR-DF	TGCGCAAGCTTCTGCGACT
Farred-F	CATGCCATGGTTGGTGAAGATAGTGT
Farred-R	CCCAAGCTTTTAACTATGACCCAATTTAC
mleRS1-BglII-F	GGAAGATCTAGTGTTACCTCCATGAAACGGTTGA
mleRS1-NcoI-R	CATGCCATGGAATCAACACTCCTTCTAAGTGAGCG
16S rRNA RT-F	GCACCGAGATTCAACATGG
16S rRNA RT-R	CTCACCAACTAGCTAATACGCC
mleS RT-F	AAGCCGACTGATCTGGCTAA
mleS RT-R	GGATTGACAAACAGCGCACT
mleT RT-F	CGCTTTCTTACTGGTTTGGTT
mleT RT-R	CTGATTTCTTCTGGGTTGGAT
maeP RT-F	ACCTAATAACCAACCCAACG
maeP RT-R	TCAACGAGTCCGCAGAAA
	

### Construction of the transcription fusion vector

The far-red fluorescent protein gene, originally derived from *Entacmaea quadricolor* (GenBank accession no. KF419293.1), was utilized to evaluate promoter activity. The transcription fusion vector was constructed as previously described (23). Briefly, the *mleST* promoter sequence, mleRS1, was amplified from *Lc. paracasei* L9 and cloned into plasmid pNZ-Farred to obtain the recombinant reporter plasmid, pNZRS. Then, the plasmid was transformed into *L. lactis* NZ9000 by electroporation. After sequencing, the recombinant plasmid was introduced into the *Lc. paracasei* L9 or *ΔmleR* mutant. Then, the positive strains were inoculated into CDM, CDM + 0.22% TDCA, CDM + 0.22% GCA, CDM + 0.22% TCA, CDM + 0.08% GDCA, CDMM, CDMM + 0.22% TDCA, CDMM + 0.22% GCA, CDMM + 0.22% TCA, CDMM + 0.08% GDCA at 37°C for 14 h. The cultures were washed and resuspended in phosphate-buffered saline with pH 7.2, and then diluted to an OD_600_ of 0.8. The fluorescence intensity of bacteria was examined under a confocal laser scanning microscope (Zeiss LSM 780, Carl Zeiss, Germany) with an excitation wavelength of 588 nm and an emission wavelength of 635 nm.

### Quantification of intracellular L-malic acid content by HPLC

The L-malic acid concentration was quantitatively analyzed by high-performance liquid chromatography (HPLC, LC-20AD, Shimadzu Corporation, Kyoto) using an Ultimate AQ-C18 column (4.6 × 250 mm, 5 μm, Aminex HPX-87H) (Welch Technology, Shanghai, China) and an SPS-20A UV-VIS detector (Shimadzu Corporation, Kyoto, Japan). When L9 was grown in CDMM or CDMM + 0.08% GDCA at 37°C for 6 and 10 h, the samples were centrifuged at 8,000 rpm for 10 min, and then the supernatant was filtered through a 0.22-μm Millipore filter. The L-malic acid was separated with the mobile phase of 20 mM Na_2_HPO_4_ (pH 2.9): methanol (99:1 vol/vol) mixture, at a flow rate of 0.7 mL/min. The standard curve for L-malic acid was generated from standard solutions of L-malic acid (0.00, 0.3125, 0.625, 1.25, 2.50, and 5.00 g/L) using the same procedure. The intracellular L-malic acid content was calculated by subtracting the detected extracellular content from the added L-malic acid and then normalized to the viable cell count, which was determined by plate colony counting. The L-malic acid concentration in CDMM without bacterial inoculation was used as a control to determine the content and verify the accuracy of this method.

### Bile salt stress assays

The MLE pathway was positively regulated by the transcriptional regulator, MleR. The deletion of the *mleR* gene could abolish MLE pathway activity. In this study, the *ΔmleR* deletion mutant was constructed by double-crossover recombination, as previously described (23). To analyze the bile tolerance of the wild-type and *ΔmleR* mutant of *Lc. paracasei* L9, the strains were grown in both CDMM and CDM + 0.08% GDCA + 5 g/L L-malic acid at 37°C. The growth of bacteria was measured using a spectrophotometer (GENESYS 30, Thermo Fisher Scientific, Waltham, MA) at a wavelength of 600 nm. The growth curves were obtained by plotting the values of OD_600 nm_ at 2 h intervals. Three biological replicates were performed for each experiment, and the data were expressed as the mean ± standard error (SD).

### Statistical analysis

Three biological replicates were performed for each experiment, and the data are expressed as the mean ± standard deviation (SD). All data were analyzed using GraphPad Prism 6 software. A Student’s *t*-test was used to compare statistical differences between two groups. For comparisons among more than two groups (e.g., the four culture conditions), one-way analysis of variance (ANOVA) was considered statistically significant.

## RESULTS

### GDCA synergized with L-malic acid to enhance the expression of MLE pathway by RT-qPCR

**Fig 1:**
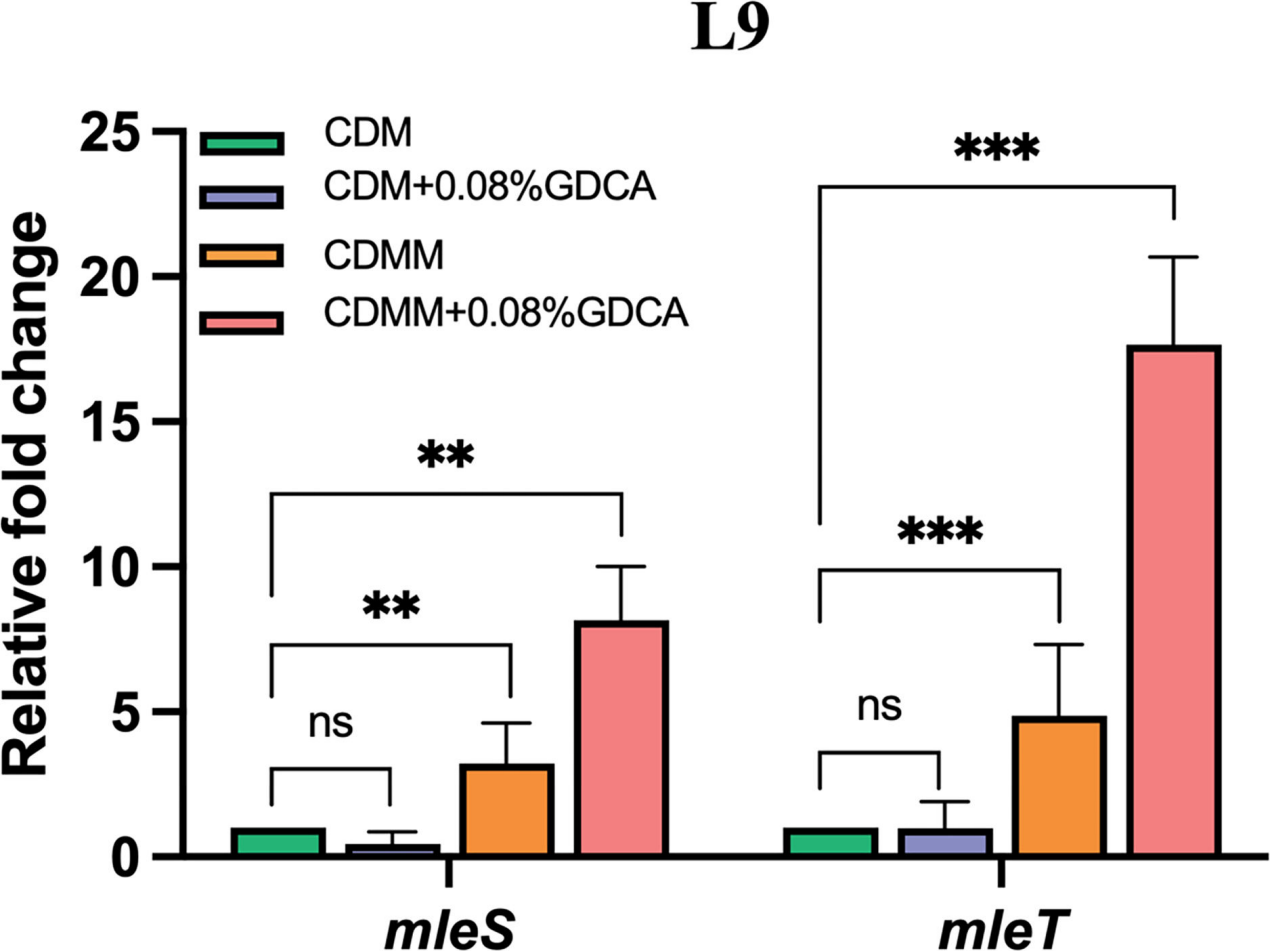
The relative expression of *mleS* and *mleT* genes in the *Lc. paracasei* L9. The fold changes compared to the CDM control. Values were normalized using the 16S rRNA gene as an internal control. Data are reported as the mean ± SD from three independent experiments (*n* = 3). Statistical significance was determined by one-way ANOVA relative to the CDM control (**P < 0.01, ***P < 0.001; ns, not significant).

To determine the response of the MLE pathway to bile salt stress, the transcription levels of *mleS* and *mleT* genes were determined by RT-qPCR. As shown in Fig. 1 and Table S1, when *Lc. paracasei* L9 was grown in CDM alone or CDM supplemented with 0.08% GDCA (CDM + 0.08% GDCA), no significant changes in *mleS* or *mleT* transcription were observed. However, compared to the CDM control, supplementation with 5 g/L L-malic acid (CDMM) significantly increased the transcript levels of *mleS* by 3.22-fold (*P* < 0.01) and *mleT* by 4.87-fold (*P* < 0.001). Notably, when 0.08% GDCA was added to CDMM (CDMM + 0.08% GDCA), the transcript levels of *mleS* and *mleT* were further increased by 8.16-fold (*P* < 0.01) and 17.67-fold (*P* < 0.001), respectively. These results demonstrated that GDCA and L-malic acid synergistically enhance the expression of the MLE pathway.

### GDCA synergizing with L-malic acid to enhance the expression of MLE pathway was further confirmed by transcriptional fusion experiments

**Fig 2:**
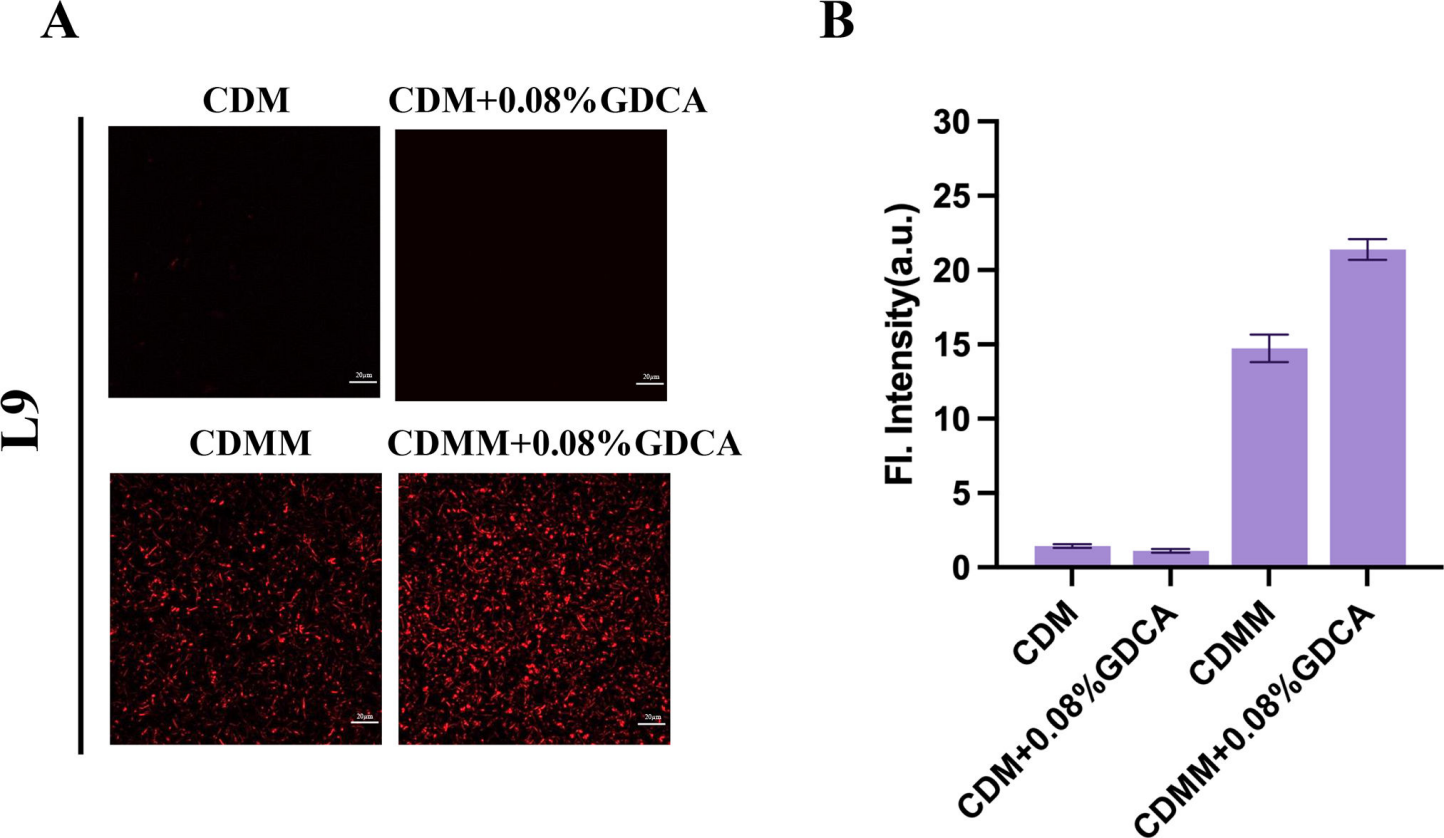
Promoter activity analysis of *P_mleST-Farred_* in *Lc. paracasei* L9 using a red fluorescent protein reporter. (**A**) Representative confocal laser scanning microscopy images showing the promoter activity of *Lc. paracasei* L9 harboring the *P_mleST-Farred_* reporter fusion. The excitation/emission wavelengths of red fluorescence protein are 588/635 nm. (**B**) Quantitative analysis of red fluorescence intensity in the *Lc. paracasei* L9 strain carrying the *P_mleST-Farred_* reporter. Data are presented as mean ± SD from three independent biological replicates (*n* = 3).

To further confirm that GDCA and L-malic acid synergistically enhance the expression of the MLE pathway, transcriptional fusion experiments were carried out in this study. As shown in Fig. 2, no fluorescence was detected when *Lc. paracasei* L9 was grown in CDM or CDM supplemented with 0.08% GDCA. The red fluorescence of bacteria was observed when L9 was cultured in CDMM and CDMM supplemented with 0.08% GDCA. Notably, the intensity of red fluorescence of *Lc. paracasei* L9 in the CDMM was significantly lower than that grown in CDMM under bile stress conditions. These findings provided further evidence that GDCA and L-malic acid synergistically enhance the expression of the MLE pathway.

To determine whether the activation of the MLE pathway by GDCA is specific, transcriptional fusion experiments were conducted to detect the promoter activity of L9 in the presence of TDCA, GCA, and TCA. As shown in Fig. S1, the addition of TDCA, GCA, and TCA to CDMM significantly increased promoter activity. These results indicated that GDCA-mediated MLE pathway activation is not bile salt-specific.

### GDCA synergistically enhanced the MLE pathway expression in a dose-dependent relationship with L-malic acid

**Fig 3:**
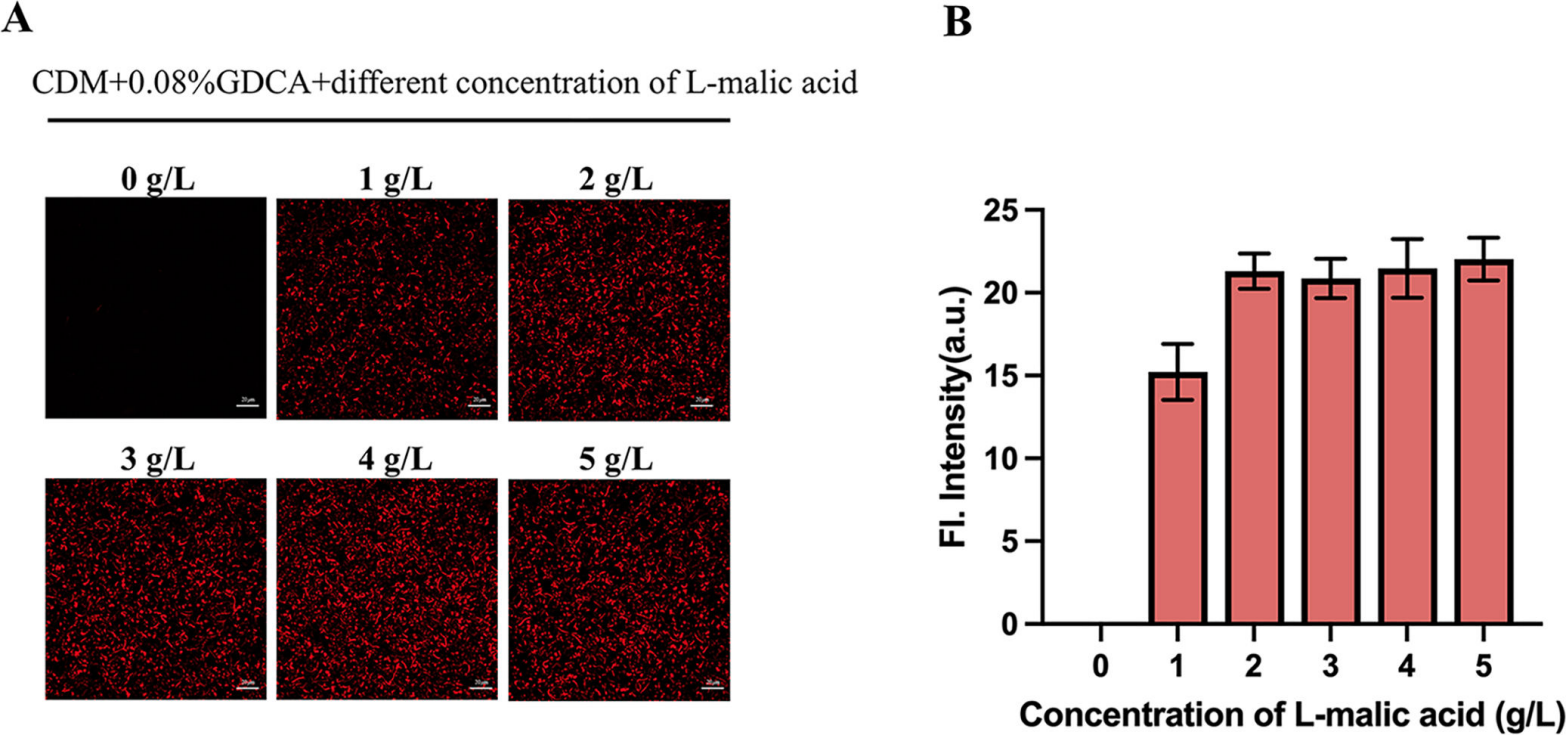
Effect of L-malic acid concentration on *P_mleST-Farred_* promoter activity in *Lc. paracasei* L9 under GDCA stress. (**A**) Expression of the *P_mleST-Farred_* transcription fusions at different concentrations of L-malic acid under GDCA stress. (**B**) The red fluorescence intensity of *Lc. paracasei* L9 strain harboring the *P_mleST-Farred_* reporter fusion. Data are presented as mean ± SD from three independent biological replicates (*n* = 3).

In order to investigate the influence of L-malic acid concentration on activation of the MLE pathway under GDCA stress, the expression of the *P_mleST-Farred_* transcription fusions was measured under 1.0, 2.0, 3.0, 4.0, and 5.0 g/L of L-malic acid. The results showed that when *Lc. paracasei* L9 was grown in CDMM supplemented with 0.08% GDCA, the fluorescence intensity increased gradually with increasing L-malic acid concentration, which reached the highest level at 2.0 g/L of L-malic acid (Fig. 3), suggesting that GDCA synergistically enhanced the MLE pathway expression in a dose-dependent relationship with L-malic acid.

### GDCA promoted intracellular transport of L-malic acid to enhance the expression of mle genes

**Fig 4:**
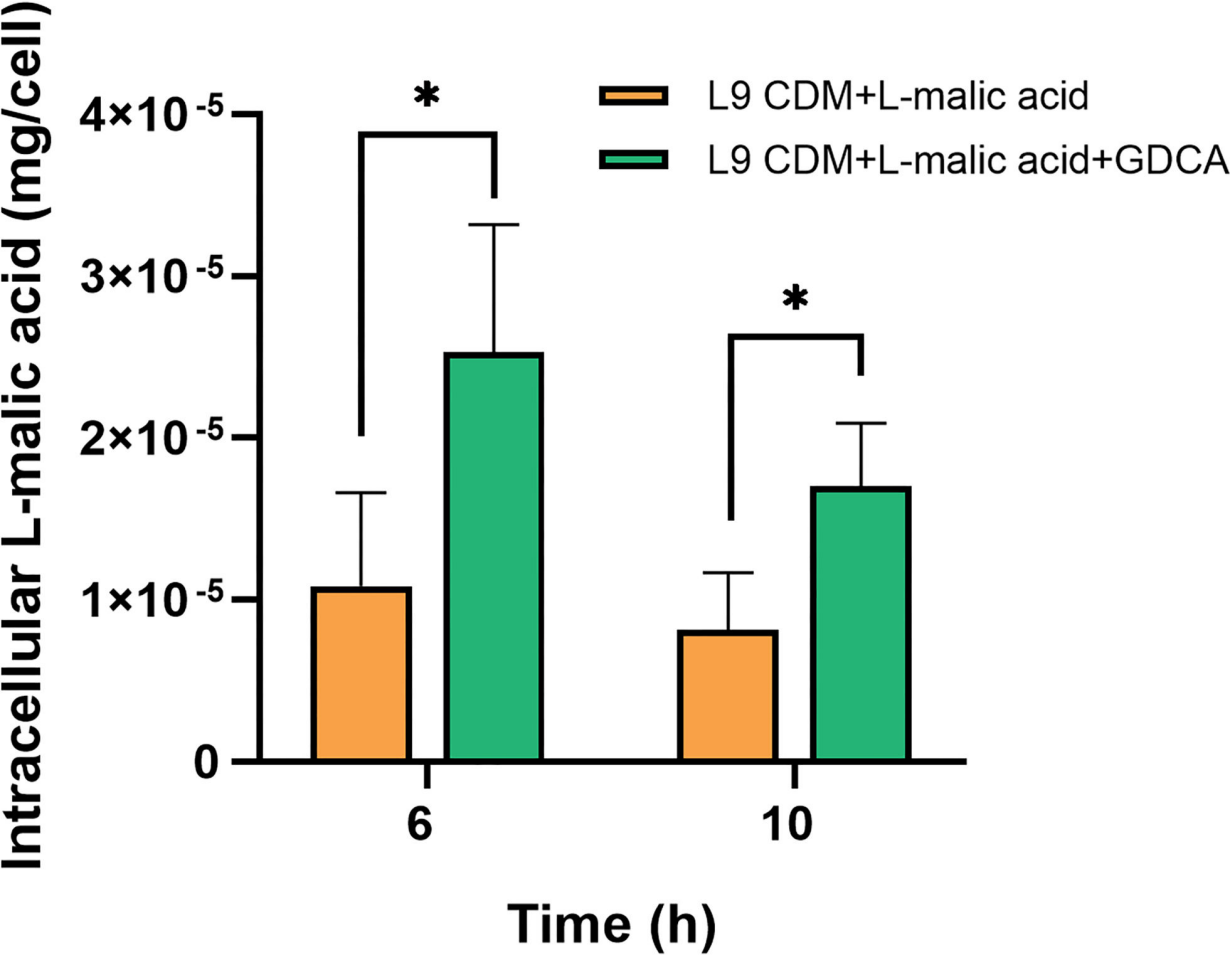
The concentration of intracellular L-malic acid in *Lc. paracasei* L9 at 6 and 10 h. Data are reported as the mean ± SD from three independent experiments (*n* = 3). Statistical significance was determined by Student’s *t*-test comparing the GDCA-supplemented group with the control group at each time point (*P < 0.05).

In order to elucidate the mechanism of GDCA synergistically enhancing the expression of the MLE pathway, the effect of GDCA on intracellular L-malic acid concentration was measured in *Lc. paracasei* L9. When L9 was cultured in CDM supplemented with 5 g/L L-malic acid, the intracellular L-malic acid concentration at 6 and 10 h was 1.08 × 10^−05^ mg per cell and 0.82 × 10^−05^ mg per cell, respectively (Fig. 4). However, the presence of GDCA significantly improved the intracellular L-malic acid concentration of the strain to 2.53 × 10^−05^ mg per cell and 1.70 × 10^−05^ mg per cell at 6 and 10 h, which was 2.33-fold and 2.09-fold higher than that of the bacteria grown in 5 g/L L-malic acid alone. These results indicated that GDCA upregulates the expression of the MLE pathway through enhancing the internal transport of L-malic acid.

### GDCA synergized with MLE pathway to alleviate self-toxicity fo*r Lc. paracasei* L9

**Fig 5:**
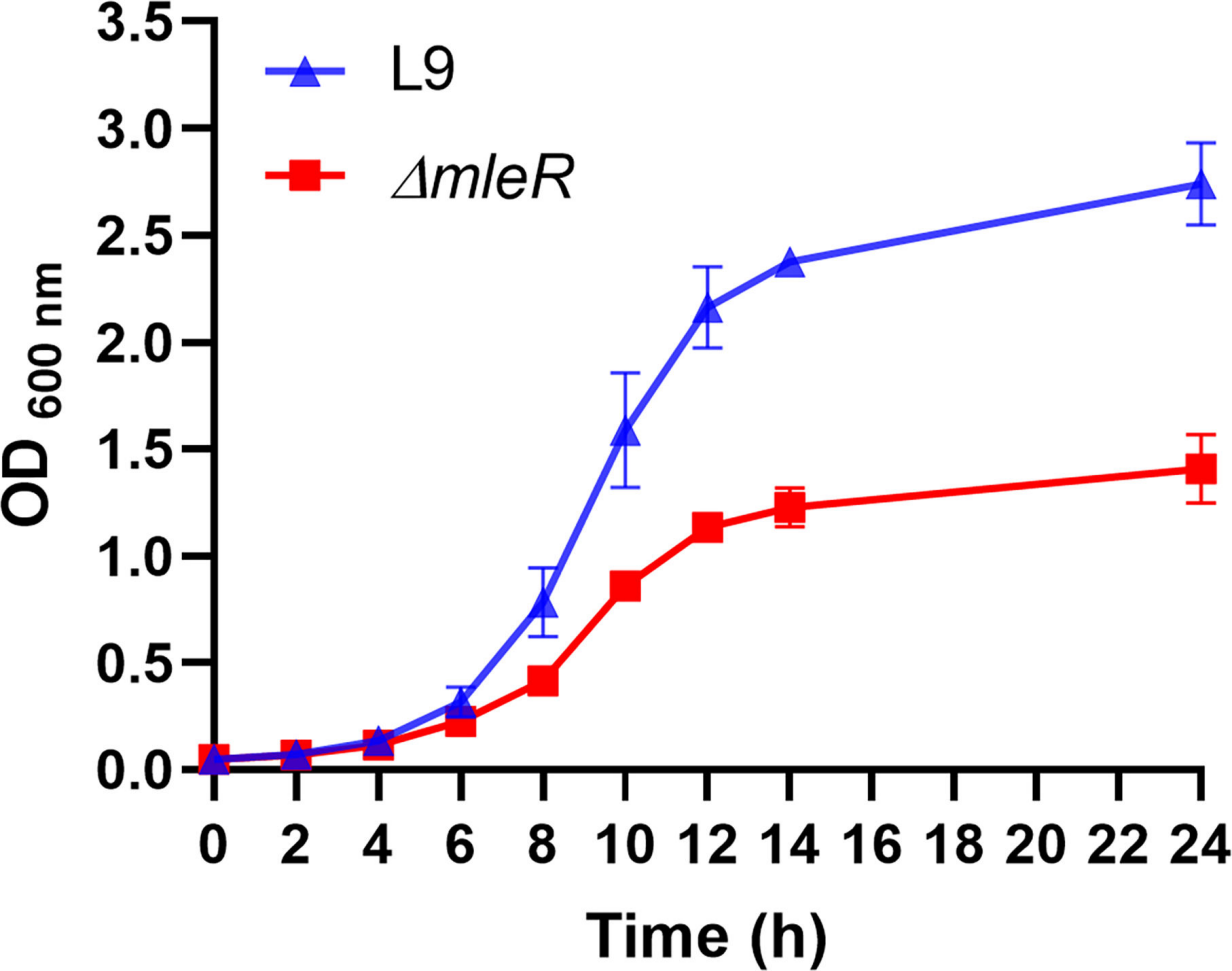
Growth curves of *Lc. paracasei* L9 wild type and *ΔmleR* under CDM + 0.08% GDCA + 5 g/L L-malic acid. The results were obtained from at least three independent experiments. The error bars correspond to the standard error (SD).

The MLE pathway is positively regulated by the transcriptional activator MleR. To investigate whether GDCA synergizes with the MLE pathway to enhance bile salt tolerance in *Lc. paracasei* L9, the *mleR*-deleted strain (*ΔmleR)* was constructed. In the absence of GDCA, deletion of *mleR* did not significantly alter the OD_600 nm_ of the strain grown in CDMM compared with the wild-type strain (Fig. S2). However, when cultured in CDMM supplemented with 0.08% GDCA, the wild-type strain reached an OD_600 nm_ of 2.38 at 14 h, whereas the OD_600 nm_ of the *ΔmleR* mutant decreased significantly to 1.23 (Fig. 5). These results indicated that GDCA and L-malic acid synergize via the MLE pathway to enhance bile salt tolerance for *Lc. paracasei* L9.

## DISCUSSION

This study elucidates a novel synergistic mechanism by which GDCA cooperates with L-malic acid to regulate the MLE pathway. *Lc. paracasei* L9 has two pathways for L-malate degradation, including the MLE and the malic enzyme (ME) pathways. In the ME pathway, malic acid as a carbon source can be converted into pyruvate (24, 25). However, the ME pathway could be repressed by carbon catabolite repression CcpA in the presence of glucose (19, 20). Since CDM contains glucose, the ME pathway is naturally repressed by carbon catabolite repression. As expected, the expression levels of *maeE* (encoding the malic enzyme) and *maeP* (encoding the malate transporter) remained extremely low and showed no significant changes under any of the tested conditions (Fig. S3 and Table S2). These results confirm that the ME pathway is not involved in the GDCA tolerance response. Through RT-qPCR and transcriptional fusion experiments, we demonstrated that GDCA significantly upregulates *mleS* and *mleT* gene expression in the presence of L-malic acid, with this synergistic effect exhibiting L-malic acid concentration dependence and reaching its peak at 2.0 g/L. HPLC quantitative analysis further revealed that GDCA enhances MLE pathway activity by promoting intracellular L-malic acid transport. The significantly reduced bile salt tolerance of the *ΔmleR* deletion strain confirmed the central role of MleR-mediated transcriptional regulation in this synergistic effect. These findings clarify the molecular mechanism by which bile salt stress signals indirectly activate protective metabolic pathways through modulating metabolite transport efficiency, providing new insights into the understanding of multi-stress adaptation strategies in lactic acid bacteria.

**Fig 6:**
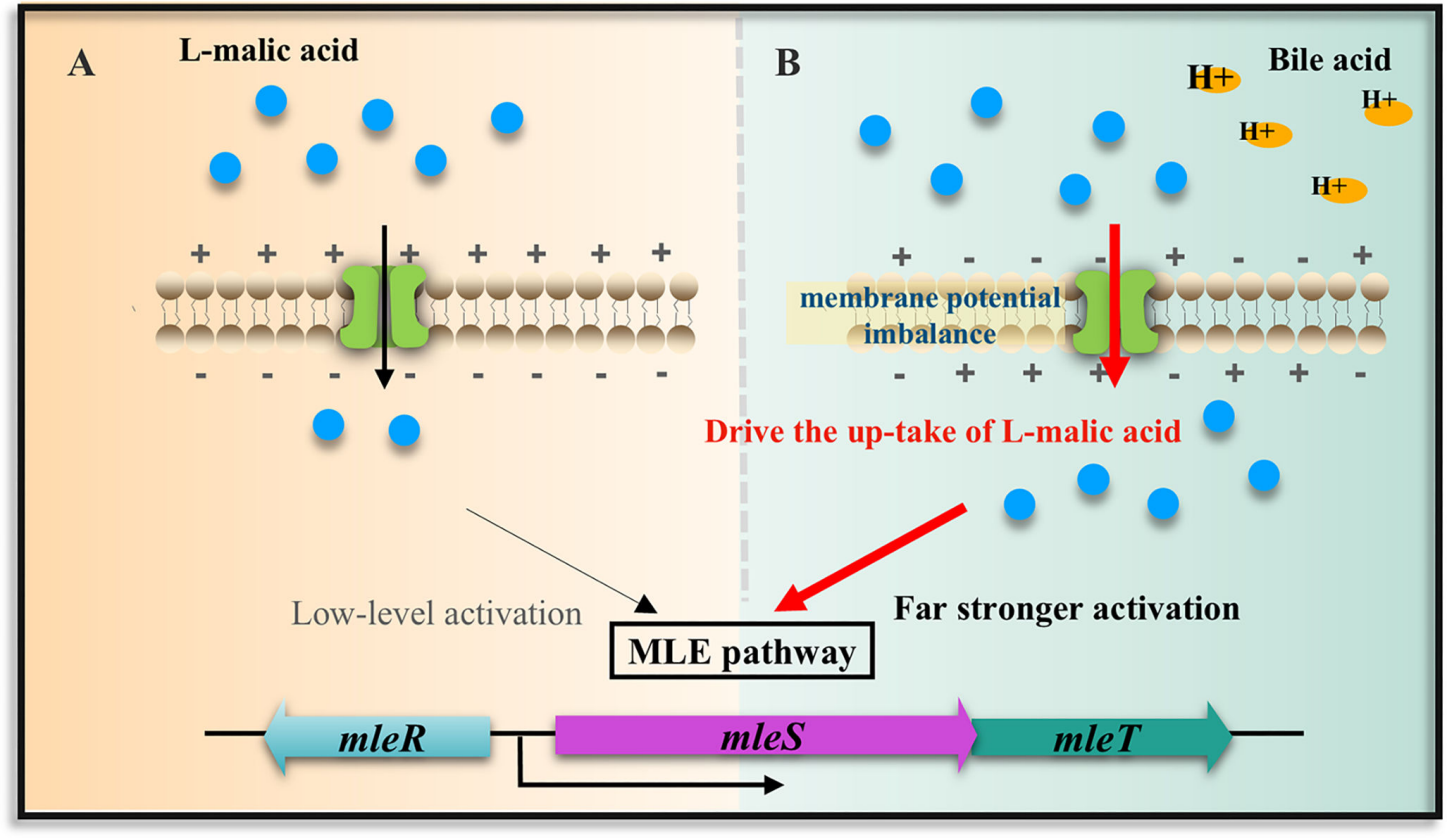
Illustration of the mechanism of GDCA stress environment induces the increase of cytosolic L-malic acid. (**A**) *Lc. paracasei* L9 under L-malic acid without bile stress. (**B**) *Lc. paracasei* L9 under L-malic acid with bile salt stress.

The MLE pathway serves as a critical metabolic module for lactic acid bacteria to cope with environmental stresses (26, 27), consuming intracellular H^+^ through L-malic acid decarboxylation to produce L-lactic acid and CO₂, thereby elevating cytoplasmic pH while generating proton motive force to drive ATP synthesis (20). Previous studies have shown that the MLE pathway in *Lc. paracasei* L9 responds not only to acid stress but also participates in bile salt stress adaptation (16, 23). This study first reports that bile salt stress signals (GDCA) can enhance the activation of the MLE pathway by the inducer L-malic acid. Based on our results, we proposed a novel bile response model of the MLE pathway in *Lc. paracasei* L9 (Fig. 6). L-malic acid was taken into cells in an anionic form by the L-malic acid transporter (28), while the efficiency of internal L-malic acid transport was limited by an internally negative membrane potential under normal physiological conditions (Fig. 6A). GDCA inserts into the phospholipid bilayer and inverts membrane potential (29), which unexpectedly facilitates L-malic acid influx (Fig. 6B). HPLC analysis also confirmed that intracellular L-malic acid concentration increased significantly in the presence of GDCA. In our previous study, we explored the complex regulatory mechanism of the MLE pathway by the LysR type regulator, MleR, in *Lc. paracasei* L9 (23). The transcriptional regulator MleR was identified to positively regulate the MLE pathway by binding −80 to −61 upstream of the transcription start site of the *mleST* operon. The *mleR* knockout inactivates *mleS* and *mleT* of the MLE pathway (Fig. S4 and Table S3). Under bile stress, the elevated L-malic acid then acts as the co-inducer of MleR, resulting in the activated transcription of the target gene *mleST* (19). This mechanism transforms membrane damage into a metabolic defense advantage without requiring direct GDCA sensing by MleR, representing an efficient substrate-induced regulatory strategy for stress adaptation.

Our finding also reveals the adaptive strategy of intestinal lactic acid bacteria in response to complex stress environments. Bile salt concentrations in the human intestine can reach 10 mM during digestion (30, 31), while dietary sources of L-malic acid provide utilizable substrates for intestinal microbiota (32, 33). *Lc. paracasei* L9 senses membrane perturbation signals mediated by bile salt and exploits this physicochemical effect to enhance L-malic acid influx, rapidly activating the MLE pathway to achieve self-detoxification. On one hand, consuming cytoplasmic H^+^ through decarboxylation reactions alleviates bile salt-induced intracellular acidification. On the other hand, maintaining membrane potential reduces further bile salt infiltration (34). This adaptive strategy of converting a toxic challenge into a protective response reflects the evolutionary refinement of long-term host and microbe interactions (35, 36). The existence of this synergistic mechanism may explain the relatively high abundance of *Lc. paracasei* L9 in the intestine of centenarians, as its efficient membrane metabolism coupled regulatory system confers competitive advantages in high bile salt environments.

### Conclusion

Bile stress tolerance is a crucial property of probiotics for surviving in the GIT. The MLE pathway could enhance the GDCA tolerance of *Lc. paracasei* L9. However, the regulatory mechanism of the MLE pathway by MleR in response to bile salt stress remains unclear. In this study, we found that GDCA could synergistically enhance the expression of the MLE pathway in a dose-dependent relationship with L-malic acid. Based on our findings, we proposed a novel mechanism that GDCA can enhance the concentration of intracellular inducer L-malic acid, an effector for MleR to activate *mleST* cluster transcription, which further increases the expression of the MLE pathway to respond to bile stress. These findings provide a novel protective mechanism mediated by the MleR regulator for coping with bile stress in lactobacilli.

## SUPPLEMENTAL MATERIAL

Supplemental material aem.00639-26-s0001.docx Fig. S1 to S4; Tables S1 to S3.

ASM does not own the copyrights to Supplemental Material that may be linked to, or accessed through, an article. The authors have granted ASM a non-exclusive, world-wide license to publish the Supplemental Material files. Please contact the corresponding author directly for reuse.
