# Supplementary material for: Glycodeoxycholic acid synergizes with L-malic acid to upregulate the malolactic enzyme pathway to alleviate self-toxicity in Lacticaseibacillus paracasei L9
Source: Appl Environ Microbiol. 2026 Jun 18;92(7):e00639-26. doi: 10.1128/aem.00639-26 (PMC13390482; doi:10.1128/aem.00639-26)
Supplement: Supplemental material — Fig. S1 to S4; Tables S1 to S3. [file aem.00639-26-s0001.docx]

***Supporting information***

**Glycodeoxycholic acid synergizes with L-malic acid to up-regulate the malolactic enzyme pathway to alleviate self-toxicity in *Lacticaseibacillus paracasei* L9**

Ran Huan^a^, Xin Feng^b^, Yaxin Gu^b^, Junzhu Li^b^, Jianping Cai^a^, Zhengyuan Zhai^b^, Yanling Hao^c*^

^a^ Department of Basic Innovation Research, Beijing Hospital, National Center for Gerontology; National Clinical Research Center for Gerontology; The Key Laboratory of Geriatrics of NHC; Beijing Key Laboratory of Aging Mechanism and Intervention Research on Aging-Related Diseases; Institute of Geriatric Medicine, Chinese Academy of Medical Sciences, P.R. China

^b^ College of Food Science and Nutritional Engineering, China Agricultural University, Beijing, China

^c^ Key Laboratory of Precision Nutrition and Food Quality, Department of Nutrition and Health, China Agricultural University, Beijing, China

***Corresponding author**: Yanling Hao

***Email**: [haoyl@cau.edu.cn](mailto:haoyl@cau.edu.cn)

**Running title: GDCA synergizes with L-malic acid to relieve self-toxicity**

***Supplemental figures***

*
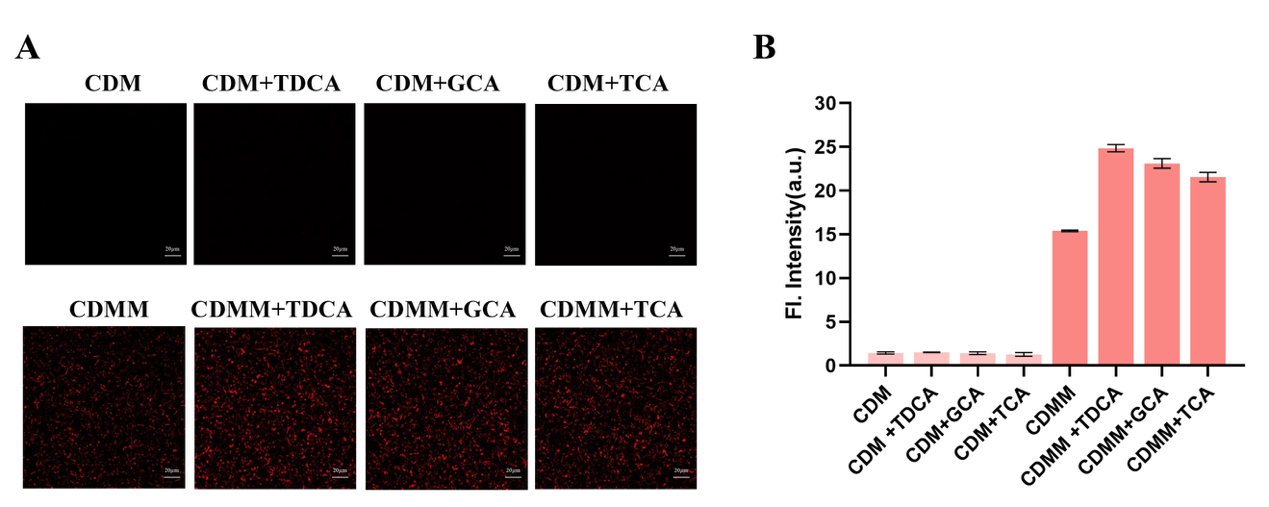
*

**Figure S1.** Influence of individual bile salts TDCA, GCA and TCA on promoter activity of *mleST* by confocal laser scanning microscope. (A) The promoter activities of *P_mleST_* in the *Lc. paracasei* L9 under TDCA, GCA and TCA. (B) The red fluorescence intensity of *Lc. paracasei* L9 strain harboring the *P_mleST-Farred_* reporter fusion. Data are reported as the mean ± SD from three independent experiments (n>3).


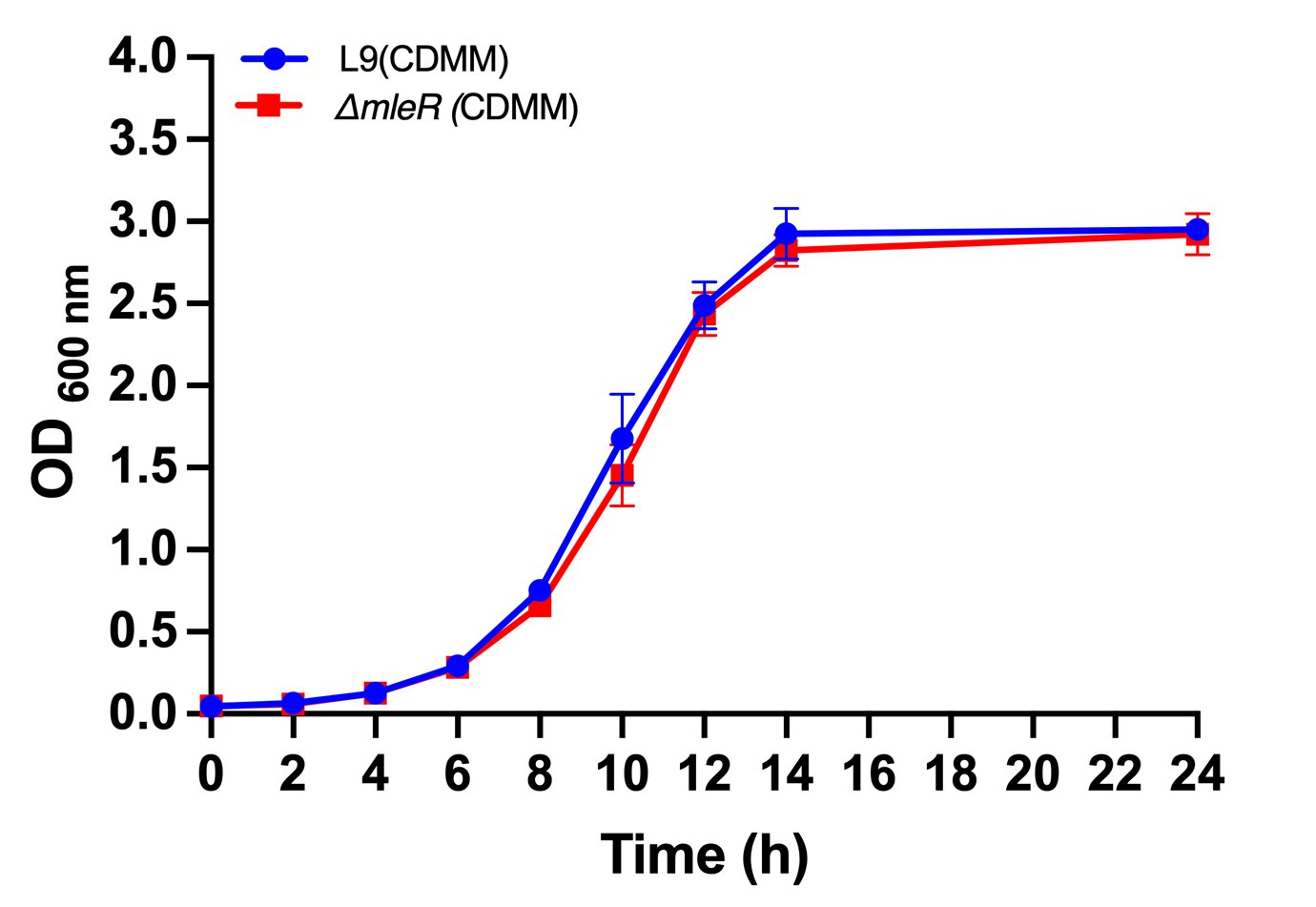


**Figure S2.** Growth curves of *Lc. paracasei* L9 wild-type and *ΔmleR* under CDM+5 g/L L-malic acid. The results were obtained from at least three independent experiments. Data are reported as the mean ± SD from three independent experiments (n=3).

*
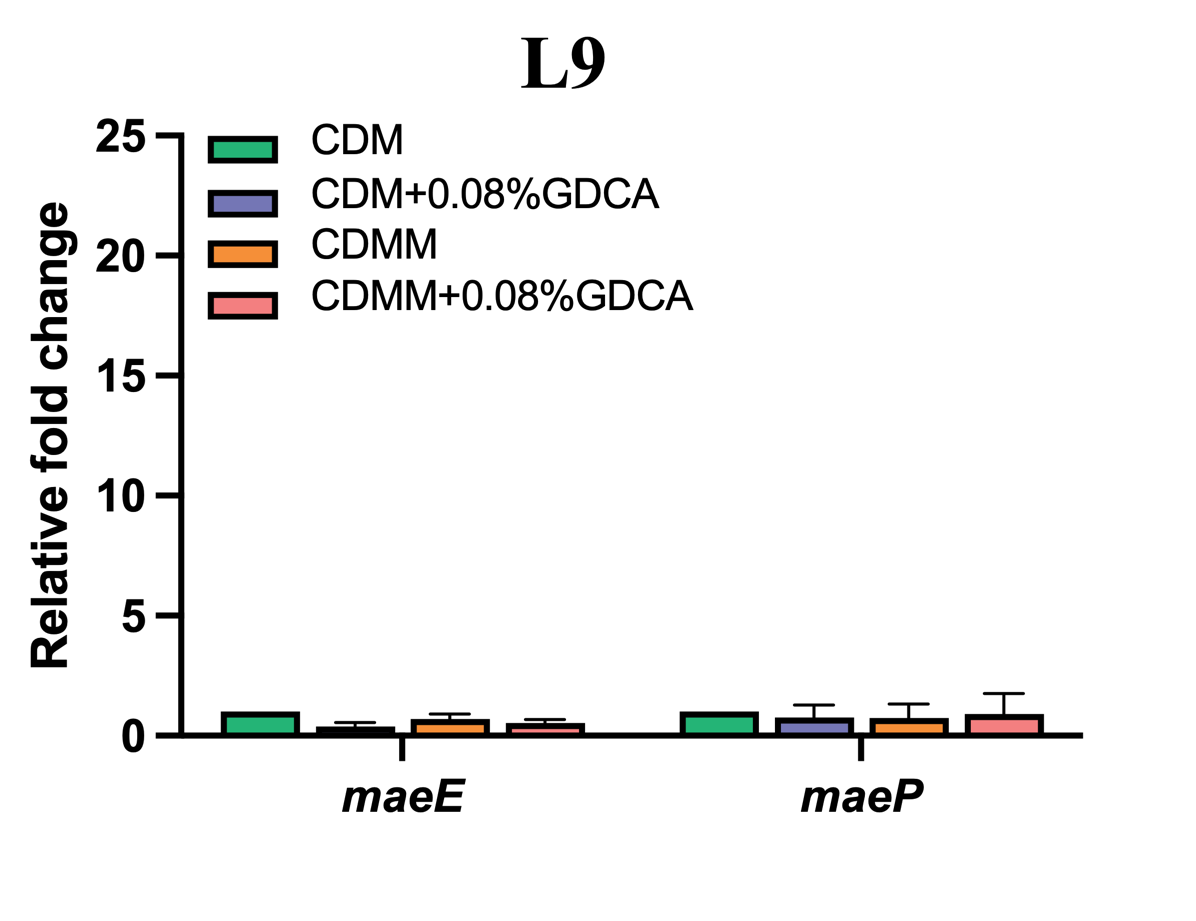
*

**Figure S3.** The relative expression of *maeE* and *maeP* genes in the *Lc. paracasei* L9*.* The fold changes compared to CDM. Values were normalized using the 16S rRNA gene as an internal control. Data are reported as the mean ± SD from three independent experiments (n=3)*.*

*
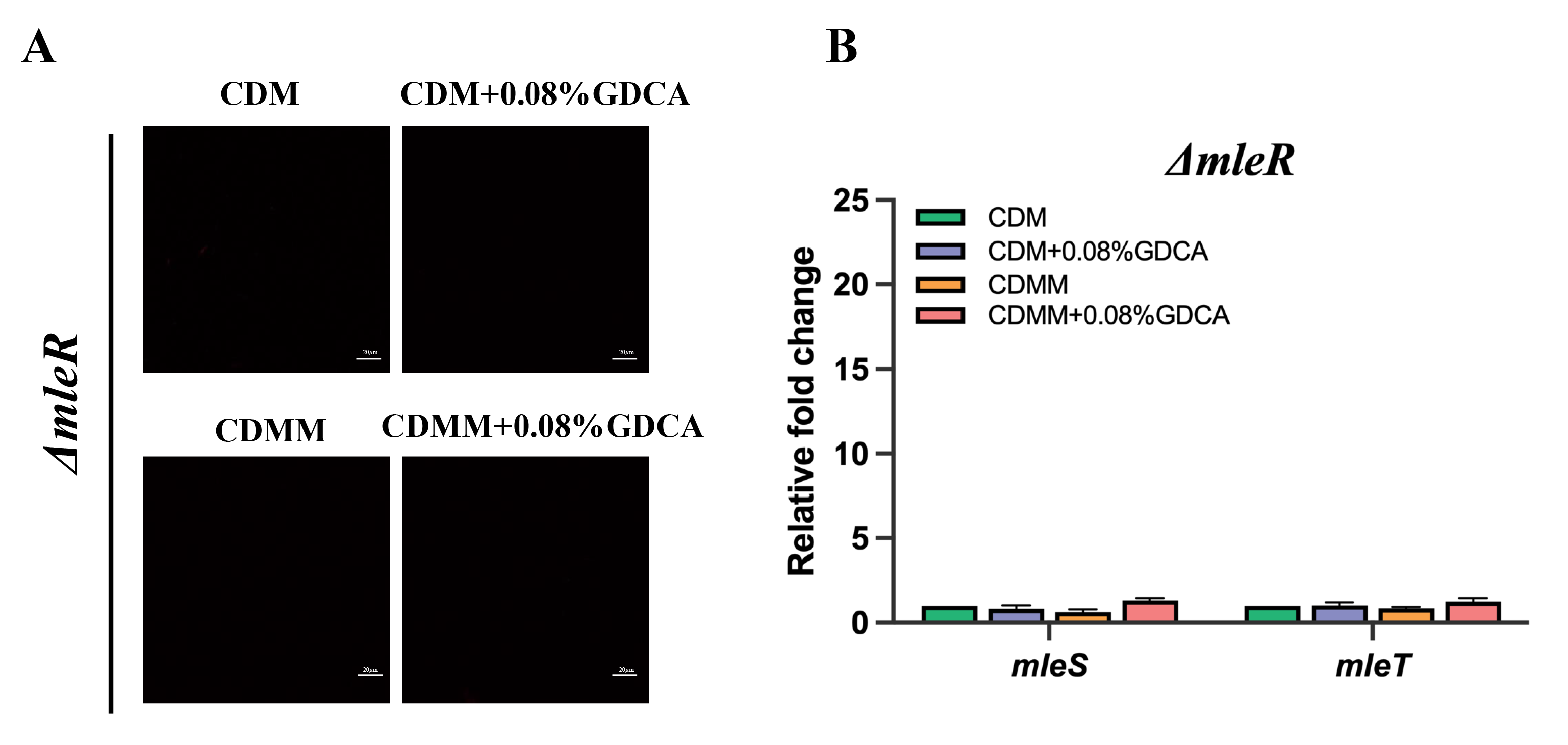
*

**Figure S4.** The transcriptional regulator MleR regulates the expression of the *mleS* and *mleT* genes in the *Lc. paracasei* L9. (A) The promoter activities of *P_mleST_* in the *ΔmleR* deletion strain followed by confocal laser scanning microscopy. The excitation/emission wavelengths of red fluorescence protein at 588/635 nm. (B) The relative expression of *mleS* and *mleT* genes in the *ΔmleR* deletion strain*.* The fold changes compared to CDM. The results were obtained from three independent experiments. The error bars correspond to the standard error (SD).

| **Group** | **Gene** | **Rep1** | **Rep2** | **Rep3** | **ΔCt-1** | **ΔCt-2** | **ΔCt-3** | **ΔΔCt-1** | **ΔΔCt-2** | **ΔΔCt-3** | **RE1** | **RE2** | **RE3** |
| --- | --- | --- | --- | --- | --- | --- | --- | --- | --- | --- | --- | --- | --- |
| CDM (control) | 16S rRNA | 15.95 | 16.02 | 16.07 | — | — | — | — | — | — | — | — | — |
| CDM (control) | mleS | 22.28 | 22.35 | 22.31 | 6.33 | 6.33 | 6.24 | 0.00 | 0.00 | 0.00 | 1.00 | 1.00 | 1.00 |
| CDM (control) | mleT | 23.46 | 23.53 | 23.49 | 7.51 | 7.51 | 7.42 | 0.00 | 0.00 | 0.00 | 1.00 | 1.00 | 1.00 |
| CDM+0.08%GDCA | 16S rRNA | 16.04 | 15.93 | 16.09 | — | — | — | — | — | — | — | — | — |
| CDM+0.08%GDCA | mleS | 22.50 | 24.27 | 24.97 | 6.46 | 8.34 | 8.88 | 0.13 | 2.01 | 2.64 | 0.92 | 0.25 | 0.16 |
| CDM+0.08%GDCA | mleT | 22.52 | 24.91 | 24.38 | 6.48 | 8.98 | 8.29 | -1.03 | 1.47 | 0.87 | 2.04 | 0.36 | 0.55 |
| CDMM | 16S rRNA | 15.98 | 16.05 | 15.92 | — | — | — | — | — | — | — | — | — |
| CDMM | mleS | 20.24 | 20.44 | 21.47 | 4.26 | 4.39 | 5.55 | -2.07 | -1.94 | -0.69 | 4.21 | 3.84 | 1.62 |
| CDMM | mleT | 20.77 | 20.99 | 22.30 | 4.79 | 4.94 | 6.38 | -2.72 | -2.57 | -1.04 | 6.59 | 5.94 | 2.06 |
| CDMM+0.08%GDCA | 16S rRNA | 16.06 | 15.97 | 16.11 | — | — | — | — | — | — | — | — | — |
| CDMM+0.08%GDCA | mleS | 19.39 | 18.97 | 19.67 | 3.33 | 3.00 | 3.56 | -3.00 | -3.33 | -2.68 | 7.98 | 10.09 | 6.42 |
| CDMM+0.08%GDCA | mleT | 19.18 | 19.56 | 19.45 | 3.12 | 3.59 | 3.34 | -4.39 | -3.92 | -4.08 | 20.99 | 15.15 | 16.86 |

*Supplemental Tables*

**Table S1.** RT-qPCR analysis of *mleS* and *mleT* gene expression in *Lc. paracasei* L9 wild-type under different culture conditions.

All Ct values and derived parameters are presented as raw data. The experiment was performed with three independent biological replicates (n=3).

**Table S2.** RT-qPCR analysis of *maeE* and *maeP* gene expression in *Lc. paracasei* L9 under different culture conditions.

| **Group** | **Gene** | **Rep1** | **Rep2** | **Rep3** | **ΔCt-1** | **ΔCt-2** | **ΔCt-3** | **ΔΔCt-1** | **ΔΔCt-2** | **ΔΔCt-3** | **RE1** | **RE2** | **RE3** |
| --- | --- | --- | --- | --- | --- | --- | --- | --- | --- | --- | --- | --- | --- |
| CDM (control) | 16S rRNA | 15.94 | 16.01 | 16.06 | — | — | — | — | — | — | — | — | — |
| CDM (control) | maeE | 21.86 | 22.32 | 22.57 | 5.92 | 6.31 | 6.51 | 0.00 | 0.00 | 0.00 | 1.00 | 1.00 | 1.00 |
| CDM (control) | maeP | 21.63 | 22.19 | 22.21 | 5.69 | 6.18 | 6.15 | 0.00 | 0.00 | 0.00 | 1.00 | 1.00 | 1.00 |
| CDM+0.08%GDCA | 16S rRNA | 15.97 | 16.03 | 15.92 | — | — | — | — | — | — | — | — | — |
| CDM+0.08%GDCA | maeE | 23.65 | 24.49 | 23.28 | 7.68 | 8.46 | 7.36 | 1.76 | 2.15 | 0.85 | 0.30 | 0.23 | 0.55 |
| CDM+0.08%GDCA | maeP | 21.27 | 24.04 | 22.74 | 5.30 | 8.01 | 6.82 | -0.39 | 1.83 | 0.67 | 1.31 | 0.28 | 0.63 |
| CDMM | 16S rRNA | 16.05 | 15.92 | 16.08 | — | — | — | — | — | — | — | — | — |
| CDMM | maeE | 22.94 | 22.34 | 23.16 | 6.89 | 6.42 | 7.08 | 0.97 | 0.11 | 0.57 | 0.51 | 0.93 | 0.67 |
| CDMM | maeP | 22.55 | 23.96 | 21.76 | 6.50 | 8.04 | 5.68 | 0.81 | 1.86 | -0.47 | 0.57 | 0.28 | 1.39 |
| CDMM+0.08%GDCA | 16S rRNA | 16.07 | 15.96 | 16.10 | — | — | — | — | — | — | — | — | — |
| CDMM+0.08%GDCA | maeE | 23.42 | 22.99 | 23.31 | 7.35 | 7.03 | 7.21 | 1.43 | 0.72 | 0.70 | 0.37 | 0.61 | 0.62 |
| CDMM+0.08%GDCA | maeP | 24.13 | 22.79 | 21.35 | 8.06 | 6.83 | 5.25 | 2.37 | 0.65 | -0.90 | 0.19 | 0.64 | 1.87 |

All Ct values and derived parameters are presented as raw data. The experiment was performed with three independent biological replicates (n=3).

**Table S3.** RT-qPCR analysis of *mleS* and *mleT* gene expression in *ΔmleR* deletion mutant under different culture conditions.

| **Group** | **Gene** | **Rep1** | **Rep2** | **Rep3** | **ΔCt-1** | **ΔCt-2** | **ΔCt-3** | **ΔΔCt-1** | **ΔΔCt-2** | **ΔΔCt-3** | **RE1** | **RE2** | **RE3** |
| --- | --- | --- | --- | --- | --- | --- | --- | --- | --- | --- | --- | --- | --- |
| CDM (control) | 16S rRNA | 15.93 | 16.02 | 16.08 | — | — | — | — | — | — | — | — | — |
| CDM (control) | mleS | 22.36 | 22.30 | 22.41 | 6.43 | 6.28 | 6.33 | 0.00 | 0.00 | 0.00 | 1.00 | 1.00 | 1.00 |
| CDM (control) | mleT | 23.52 | 23.45 | 23.58 | 7.59 | 7.43 | 7.50 | 0.00 | 0.00 | 0.00 | 1.00 | 1.00 | 1.00 |
| CDM+0.08%GDCA | 16S rRNA | 16.05 | 15.94 | 16.10 | — | — | — | — | — | — | — | — | — |
| CDM+0.08%GDCA | mleS | 22.69 | 22.22 | 23.17 | 6.64 | 6.28 | 7.07 | 0.21 | 0.00 | 0.74 | 0.86 | 1.00 | 0.60 |
| CDM+0.08%GDCA | mleT | 23.37 | 23.60 | 23.54 | 7.32 | 7.66 | 7.44 | -0.27 | 0.23 | -0.06 | 1.21 | 0.85 | 1.04 |
| CDMM | 16S rRNA | 15.96 | 16.04 | 15.91 | — | — | — | — | — | — | — | — | — |
| CDMM | mleS | 22.78 | 22.82 | 23.39 | 6.82 | 6.78 | 7.48 | 0.39 | 0.50 | 1.15 | 0.76 | 0.71 | 0.45 |
| CDMM | mleT | 23.74 | 23.81 | 23.51 | 7.78 | 7.77 | 7.60 | 0.19 | 0.34 | 0.10 | 0.88 | 0.79 | 0.93 |
| CDMM+0.08%GDCA | 16S rRNA | 16.07 | 15.95 | 16.12 | — | — | — | — | — | — | — | — | — |
| CDMM+0.08%GDCA | mleS | 22.11 | 22.00 | 21.89 | 6.04 | 6.05 | 5.77 | -0.39 | -0.23 | -0.56 | 1.31 | 1.17 | 1.47 |
| CDMM+0.08%GDCA | mleT | 23.38 | 23.23 | 23.05 | 7.31 | 7.28 | 6.93 | -0.28 | -0.15 | -0.57 | 1.21 | 1.11 | 1.48 |

All Ct values and derived parameters are presented as raw data. The experiment was performed with three independent biological replicates (n=3).
